# Retrospective Radiographic Evaluation of Ridge Dimensional Changes After Vertical Augmentation Using the Novel Wide-Head Tent Pole Screw Technique

**DOI:** 10.3390/jfb16060215

**Published:** 2025-06-09

**Authors:** Nam-Suk Yoon, Hyunsuk Choi, Hyung-Gyun Kim, Dong-Seok Sohn

**Affiliations:** 1 Department of Dentistry and Oral and Maxillofacial Surgery, Daegu Catholic University School of Medicine, Daegu 42472, Republic of Korea; ff0311@naver.com; 2 Department of Dentistry and Prosthodontics, Daegu Catholic University School of Medicine, Daegu 42472, Republic of Korea; hschoi@cu.ac.kr; 3 Department of Dentistry and Advanced General Dentistry, Daegu Catholic University School of Medicine, Daegu 42472, Republic of Korea; hgkim25@cu.ac.kr

**Keywords:** vertical ridge augmentation, guided bone regeneration, bone grafts, tenting pole screw, peri-implantitis, vertical bone defects

## Abstract

Introduction: Although significant advancements have been made in surgical techniques for reconstructing severely resorbed alveolar bone, achieving predictable regeneration remains a considerable challenge. Many conventional ridge augmentation methods require extended edentulous healing periods and multiple surgical interventions. This clinical study introduces a simplified approach to advanced ridge augmentation using a wide-head tent-pole screw (WHTPS), aimed at enhancing procedural efficiency and achieving predictable clinical outcomes. Material and Methods: Thirteen patients with severely vertically resorbed mandibular segments or completely edentulous alveolar ridges—each presenting with a minimum vertical bone defect of 5 mm—were included in this study. A single WHTPS was placed at the most severe bone defect site, followed by bone grafting and coverage with a resorbable membrane. Postoperative panoramic radiographs were taken immediately after surgery and again on the day of WHTPS removal, following a healing period of 3 to 6 months. An additional follow-up radiograph was obtained after final prosthesis placement, with an average follow-up period of 5.5 months, to assess changes in the augmented bone. Patients were monitored clinically for a period ranging from 8 to 20 months (mean: 14.9 months). Results: The average vertical bone gain immediately after surgery was 8.86 mm (SD ± 2.59 mm), while an average bone resorption of 1.49 mm (17.79%) was observed during the follow-up period. Conclusions: A single WHTPS effectively stabilized the graft material in cases of severe alveolar bone loss, thereby preventing its resorption and displacement. Further clinical studies are necessary to validate its long-term effectiveness.

## 1. Introduction

The loss of teeth not only compromises masticatory function but also negatively impacts overall well-being and facial aesthetics [1]. Dental implants are widely regarded as the gold standard for restoring oral function due to their natural appearance, functional reliability, and long-term success [2]. However, implant placement in sites with severe vertical bone deficiency remains one of the most technically challenging procedures in implant dentistry [3]. Various surgical techniques have been proposed to augment severely atrophic alveolar ridges, but many of these approaches are associated with limitations such as prolonged operative time, increased surgical morbidity, and extended edentulous healing periods [4,5,6,7,8,9,10]. In cases of significant mandibular bone loss, bone grafts are prone to rapid resorption, often due to insufficient soft tissue coverage and the resulting compressive forces on the grafted site. To address these limitations, a technique using dental implants to generate a “tenting effect” has been introduced. This method improves graft stabilization and helps maintain graft volume by lifting the soft tissue matrix [11,12,13]. When simultaneous implant placement is not feasible, small-diameter tenting pole screws have been used as an alternative to create the necessary tenting effect and facilitate vertical bone regeneration. Although simpler than other vertical augmentation techniques, this approach has limitations. Narrow-headed mini screws offer limited expansion of the soft tissue matrix, often resulting in suboptimal vertical bone gain. To compensate, multiple screws are typically required, which increases surgical complexity and operative time [14,15]. To overcome these drawbacks, a wide-head tent-pole screw (WHTPS) has been developed to enhance the tenting effect using a single device. This study aims to evaluate the clinical and radiographic effectiveness of WHTPS for vertical ridge augmentation, particularly in cases with severe vertical bone deficiency. Through a 24-month follow-up period, this study assesses the WHTPS’s capacity to maintain vertical bone height and simplify the augmentation process, validating its potential as a practical solution for challenging ridge defects.

## 2. Materials and Methods

Patient selection. This study included 13 patients treated between 2018 and 2022 at Daegu Catholic University Hospital, with approval from the Institutional Review Board (IRB No. 2024-11-002). The patients were between 44 and 71 years of age. Inclusion criteria were edentulous posterior mandibular regions with ≥5 mm vertical ridge deficiency, confirmed by cone beam computed tomography (CBCT) and panoramic radiographs. All patients required vertical ridge augmentation to support prosthetic rehabilitation using multiple splinted implants. Exclusion criteria included uncontrolled systemic conditions affecting bone healing (e.g., diabetes mellitus, osteoporosis), active infection, or history of bisphosphonate use, chemotherapy, or radiotherapy within the past year. Although smokers were informed of the potential risk of delayed healing, they were not excluded from the study. All participants provided written informed consent for both the treatment and the use of their data for research purposes.

**Figure 1:**
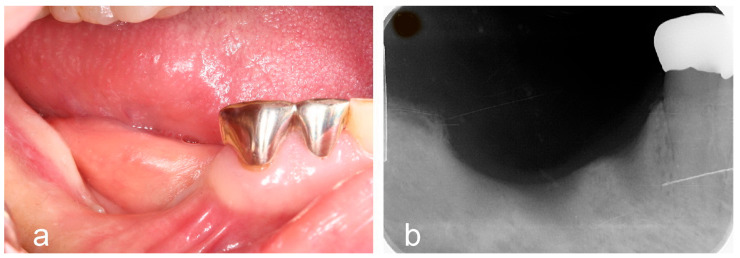
Preoperative intraoral photograph and periapical radiograph. (**a**) Clinical view showing a severe vertical soft and hard tissue defect following implant removal due to peri-implantitis. (**b**) Radiographic image confirming the presence of a substantial vertical bone defect in the edentulous ridge.

**Figure 2:**
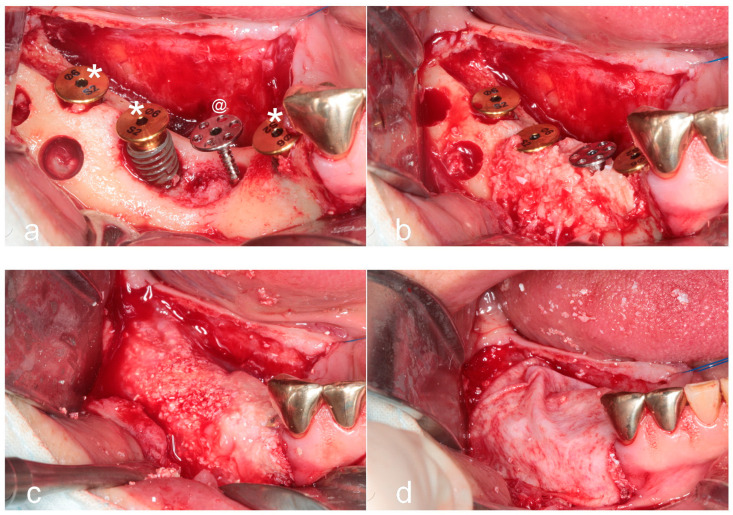
Surgical procedure. (**a**) Following elevation of full-thickness buccal and lingual flaps, an approximately 8 mm vertical bone defect was identified. A 6 mm-wide × 12 mm-long WHTPS (@) was inserted at the pontic site, and tent-pole abutments (*) were connected to each implant to support over-grafting above the implant platform. (**b**) Sticky autogenous bone, harvested from the buccal cortical bone, was grafted into the defect. (**c**) Sticky bovine bone was applied to the remaining area to enhance graft stability and volume. (**d**) A resorbable collagen membrane was placed over the graft without the use of membrane tacks or fixation sutures.

**Figure 3:**
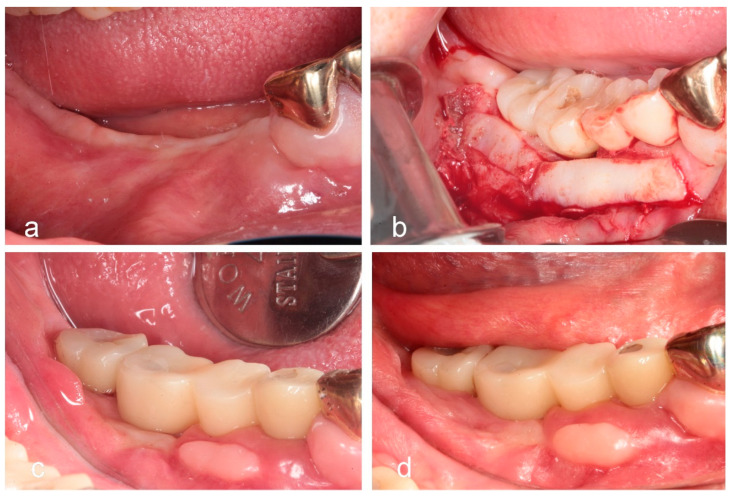
Uncovering after healing and placement of restoration. (**a**) Intraoral view taken at the time of implant uncovering, 4 months after surgery. (**b**) Sutureless free gingival graft performed to increase the width of keratinized tissue following provisional restoration delivery. (**c**) Intraoral image after placement of the final zirconia-based prosthesis. (**d**) Intraoral view after 2 years of functional loading, showing stable peri-implant soft tissue conditions.

**Figure 4:**
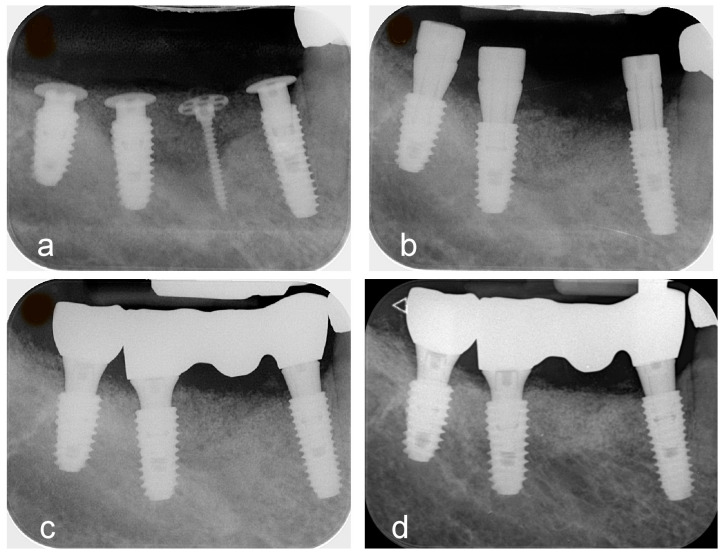
Serial radiographs. (**a**) Panoramic radiograph obtained immediately after surgery. (**b**) Radiograph taken 4 months postoperatively at the time of implant uncovering. (**c**) Baseline radiograph captured at the time of final prosthesis delivery. (**d**) Radiograph after 2 years of functional loading, demonstrating stable maintenance of the augmented ridge above the implant platform.

**Figure 5:**
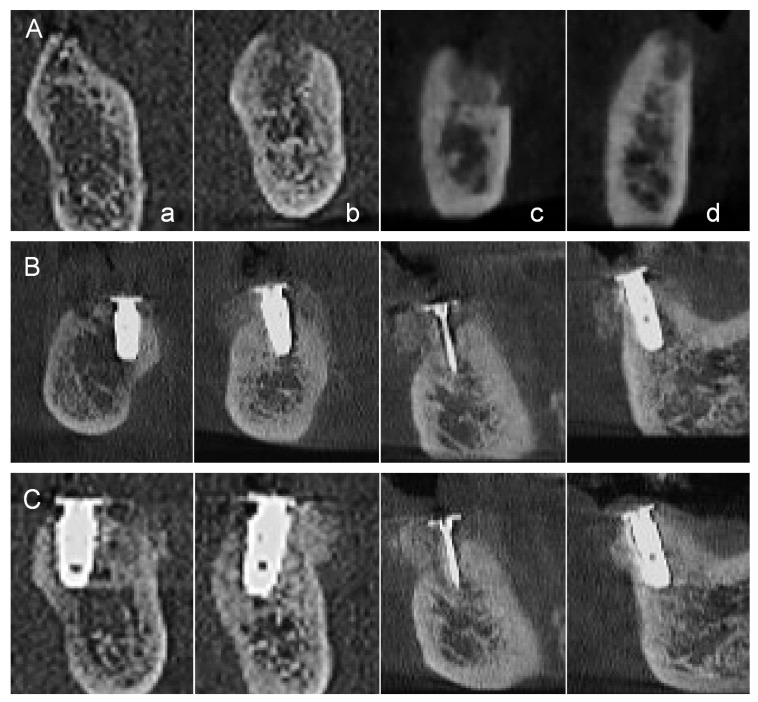
**Serial cross-sectional CBCT images.** Tooth positions: a—second molar; b—first molar; c—second premolar (WHTPS site); d—first premolar. (**A**) Preoperative scan showing vertical alveolar bone deficiency at the edentulous site. (**B**) Immediate postoperative scan following vertical ridge augmentation using a wide-head tent pole screw (WHTPS) and particulate bone graft. (**C**) Scan at 4 months postoperatively, taken on the day of uncovering, showing stable graft volume and cortical bone formation.

Surgical and restoration procedure. All surgical procedures were performed by a single experienced oral and maxillofacial surgeon under local anesthesia (2% lidocaine with 1:100,000 epinephrine), following intravenous administration of prophylactic antibiotics (Flomoxef, Flumarin^®^, Ildong Pharm, Seoul, Republic of Korea). Venous blood was collected to prepare autologous fibrin glue and concentrated growth factor (CGF) membranes for creating “Sticky Bone,” a technique first introduced by Sohn et al. [16]. This biomaterial improves handling, space maintenance, and stability of the graft, while minimizing particle migration and enhancing the healing environment. A crestal incision extending from the retromolar pad was made, accompanied by anterior and posterior vertical incisions that extended divergently beyond the mucogingival junction at a 45° angle. The lingual full-thickness flap was coronally and lingually elevated using a periosteal elevator, dissecting the periosteum and the superficial fibers of the mylohyoid muscle. The buccal flap was released via periosteal scoring with a No. 15c blade. Residual soft tissue within the defect was carefully removed. A surgical guide (BonePen Guide, Acrodent Co., Busan, Republic of Korea) was used to ensure proper implant positioning. Under-osteotomy was performed using a drill 1 mm narrower than the implant diameter to enhance primary stability. Implants (Biotem Implant Co., Seoul, Republic of Korea) were placed 2 mm subcrestally in the edentulous ridge, serving as tenting structures. To facilitate over-grafting, a 2 mm-high tenting pole abutment (SANTA^®^, Biotem Implant Co., Seoul, Republic of Korea) was connected to the implant platform and torqued to 10 Ncm. The tenting pole abutment is a specialized implant component designed to preserve vertical space during bone regeneration. It acts like a “tent pole” by supporting the overlying membrane or soft tissue, preventing collapse and enabling predictable vertical ridge regeneration. In the area of the most severe vertical defect—where implant placement was avoided to prevent inferior alveolar nerve injury—a wide-head tent-pole screw (WHTPS; SBB^®^, Biotem Implant Co., Seoul, Republic of Korea) measuring 6–8 mm in diameter and 12–14 mm in length was placed into the buccal or lingual cortical bone. The WHTPS was inserted using a 0.48-inch hex driver attached to an implant motor at 40 rpm, with its head positioned to match the height of adjacent SANTA abutments. Autogenous bone was harvested from the buccal alveolar ridge posterior to the distal implant using an ACM (Autobone Chip Maker; Neobiotec, Seoul, Republic of Korea). The harvested bone was combined with CGF-derived fibrin glue to form sticky autogenous bone. This was grafted around the exposed implant threads, WHTPS, and defect area. Sticky bovine bone was subsequently layered over the autogenous bone to enhance three-dimensional volume. The graft material was placed to fully cover both the tenting pole abutment and WHTPS. A resorbable collagen membrane was placed over the graft, followed by a CGF membrane to promote soft tissue healing. Notably, no membrane tacks or fixation sutures were used, reducing surgical time. Tension-free primary closure was achieved using non-absorbable 4-0 nylon sutures. Panoramic and CBCT scans were obtained immediately postoperatively to measure both native and augmented bone height (Figure 1, Figure 2 and Figure 3). Sutures were removed at 2 weeks. Uncovering surgery was performed after approximately 5 months of healing, during which the WHTPS was also removed. Sutures were removed 2 weeks later, and impressions for provisional restorations were taken at the same visit. The provisional restoration was delivered after one week. After 1 month of provisional use, final impressions were taken and a zirconia-based definitive prosthesis was delivered 1 week later. A panoramic radiograph was taken at this time to assess the augmented bone and evaluate resorption over 3–7 months (mean: 5.5 months). Follow-up continued for 4–6 months after final prosthesis delivery, during which sutureless free gingival grafts were performed as needed (Figure 4 and Figure 5).

**Figure 6:**
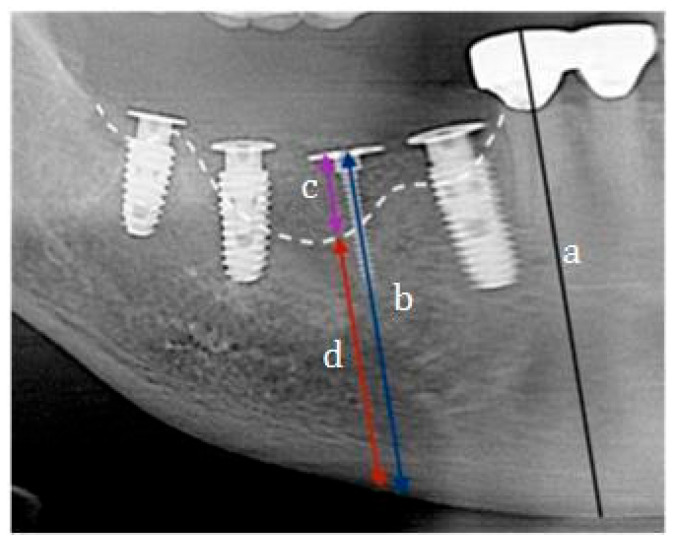
Postoperative radiographic measurement of subject (**a**) Reference line: A baseline drawn parallel to the long axis of the adjacent teeth. (**b**) Measurement 1: Total bone height measured from the lowest point of the mandible to the top of the wide-head tent-pole screw. (**c**) Measurement 2: Augmented bone height measured from the top of the tent-pole screw to the graft junction, where the native mandibular bone meets the grafted bone. (**d**) Original bone line: Pre-augmentation bone height measured from the mandibular base to the graft junction.

**Radiographic analysis.** Dimensional changes of the augmented ridge were evaluated using panoramic radiographs, following the method described by Park YH et al. [17] (Figure 6). A reference line was drawn parallel to the long axis of the adjacent teeth, with the top of the tent-pole screw serving as the central reference point. Measurement 1 represents the total bone height, defined as the distance from the lowest point of the mandible (mandibular base) to the top of the tent-pole screw.

Measurement 2 represents the augmented bone height, defined as the distance from the top of the tent-pole screw to the interface where the grafted bone meets the native mandibular bone (graft junction). Original Bone Height, an extension of Measurement 2, is defined as the distance from the graft junction (native bone) to the mandibular base, serving as the baseline height prior to augmentation. During the second and third measurement periods, only Measurement 1 was reassessed to evaluate long-term maintenance of the augmented bone height.

All measurements were performed twice by a single examiner, and the average value was used to ensure consistency. The measurement error was limited to ±0.1 mm. To minimize image distortion and improve accuracy, digital calibration tools and fixed magnification parameters were applied during panoramic radiograph acquisition. Statistical analysis was conducted using the Wilcoxon signed-rank test to evaluate differences in bone height before and after healing. This nonparametric test was chosen over the paired *t*-test due to the small sample size (*n* = 13) and the non-normal distribution of the data, which did not satisfy the assumptions required for parametric analysis.

## 3. Results

**Figure 7:**
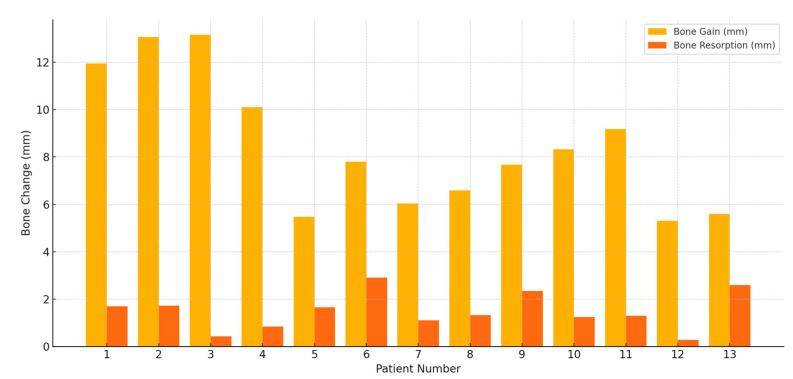
Comparison of bone gain and bone resorption.

A total of 13 patients (5 males and 8 females; mean age: 52.4 years) with severe vertical ridge deficiencies underwent vertical ridge augmentation using the wide-head tent-pole screw (WHTPS) technique. Except for one case, no soft tissue dehiscence or WHTPS exposure was observed during the healing period. Furthermore, no patients reported neurosensory disturbances such as paresthesia or dysesthesia, and no significant complications were noted aside from postoperative swelling at the recipient sites. All implants, including the one case that required secondary grafting, were successfully uncovered at the designated time point. The follow-up period ranged from 8 to 24 months (mean: 14.9 months). During this time, no implants exhibited mobility or failure, resulting in a 100% implant survival rate. Immediately after surgery, radiographic evaluations (Table 1) revealed a mean augmented bone height of 8.86 mm (range: 5.30–13.15 mm; SD: ±2.59 mm), while the preoperative bone height ranged from 5.0 to 7.2 mm (mean: 6.1 mm; SD: ±0.7 mm). At follow-up, the mean bone resorption was 1.49 mm (range: 0.28–2.91 mm; SD: ±0.78 mm), accounting for 17.79% of the initially augmented height. The trend in bone gain and resorption is illustrated in Figure 6, highlighting interpatient variability. The difference between the immediate postoperative and follow-up bone heights was statistically significant, as confirmed by the Wilcoxon signed-rank test (*p* = 0.0002) (Figure 7). Despite minor resorption, postoperative radiographs consistently demonstrated stable integration of the grafted material without signs of collapse, migration, or structural compromise. The WHTPS effectively stabilized the graft in pontic regions, supporting predictable vertical ridge augmentation. In addition, the tent-pole abutments (SANTA^®^, Biotem Implant Co., Seoul, Republic of Korea) enabled over-grafting above the implant platform, with radiographic evidence confirming well-maintained graft volume. Clinically, 12 of the 13 cases healed uneventfully, without signs of infection, inflammation, or peri-implant bone loss. One patient experienced partial wound exposure two weeks after surgery, resulting in approximately 40% graft resorption. This was successfully managed with a secondary grafting procedure performed one month later, and healing thereafter progressed without further complications.

## 4. Discussion

Vertical ridge augmentation remains one of the most technically demanding procedures in implant dentistry, primarily due to the difficulty in restoring sufficient vertical bone height for predictable implant placement. Various augmentation techniques have been developed to address this challenge, including guided bone regeneration (GBR), onlay block grafting, titanium mesh applications, distraction osteogenesis, and sandwich interpositional bone grafting. While these methods have demonstrated clinical efficacy, each presents limitations such as procedural complexity, significant resorption rates, and increased surgical morbidity [18,19]. Onlay block grafting is widely regarded as the gold standard for vertical ridge augmentation, typically involving the harvesting of autogenous bone from secondary donor sites such as the iliac crest or mandibular chin [19]. While autogenous bone offers osteogenic, osteoinductive, and osteoconductive properties, its clinical application is often limited due to significant donor site morbidity. Patients frequently experience postoperative pain, prolonged healing times, and increased surgical trauma associated with bone harvesting procedures. Additionally, block grafts are prone to resorption, with reported rates ranging from 25% to 40% within the first year, particularly in vertical ridge augmentation cases [20,21,22].

Titanium mesh has emerged as an alternative approach due to its rigid structure, which effectively maintains space for the graft material [23]. Its open architecture promotes vascularization and facilitates bone regeneration. However, mesh exposure and soft tissue irritation are common complications, potentially increasing infection risk and compromising graft success [24,25]. Distraction osteogenesis enables vertical augmentation without bone harvesting, but its use is limited by challenges in controlling vector direction and segment positioning. Furthermore, it does not address horizontal deficiencies and often requires additional procedures [26]. The sandwich technique using interpositional bone grafts can achieve 6–10 mm of vertical augmentation with reduced resorption due to its pedicled blood supply. However, it also does not address horizontal ridge deficiencies [27]. The ramus split technique has also been used as an alternative to block grafting. This technique offers long-term stability with predictable vertical and horizontal bone gain in the posterior maxilla. The use of autogenous split bone grafts and fixation pins minimizes resorption and supports reliable implant placement. However, it requires advanced surgical skills and a secondary donor site, increasing operative time and patient morbidity. Potential complications such as graft exposure or partial resorption may occur but are relatively rare [28]. GBR remains a commonly used approach due to its relative simplicity and reduced invasiveness. Studies suggest that non-resorbable membranes provide greater vertical bone gain than resorbable ones, but are associated with higher exposure risk [29]. A major drawback of staged augmentation is the delayed implant placement, which increases the total treatment time and surgical burden. The use of titanium-reinforced PTFE mesh often requires multiple fixation tacks, further extending operative time. Additionally, GBR in vertical applications is associated with early resorption rates of 20–37% within the first six months [29]. A recent meta-analysis by Tay et al. reported that vertical bone gain is significantly reduced in the presence of healing complications, particularly membrane exposure or abscess formation [30]. However, the overall incidence of such complications remained relatively low, with patient-level rates of 10–11%. These findings emphasize the importance of tension-free primary closure and uneventful healing to achieve predictable outcomes. The same analysis also found no significant differences in outcomes between simultaneous and staged implant placements, supporting simultaneous placement as a viable option to reduce treatment time and surgical steps. Vertical augmentation procedures, in general, have demonstrated an average bone gain of 4–5 mm, with complication rates ranging from 16% to 25% [31]. Compared to block bone techniques, GBR has demonstrated fewer complications but offers limited vertical augmentation capacity. To address this, a modified GBR approach incorporating tenting pole screws has been proposed to improve space maintenance and support bone regeneration [32,33]. Although vertical augmentation with mini tenting screws is less technique-sensitive than other methods, the small head diameter limits its ability to support soft tissue expansion. Consequently, multiple mini screws are often needed, increasing the risk of damage to adjacent teeth and extending surgical time. In addition, the inability to place implants simultaneously prolongs the edentulous healing period. To overcome these limitations, this study utilized a single wide-head tent-pole screw (WHTPS) in the pontic area between simultaneously placed implants. Compared to multiple tenting screw techniques, the WHTPS approach simplifies the surgical workflow, reduces the number of components, and shortens operative time while enhancing the tenting effect on soft tissue and periosteum. Additionally, the wider head of the WHTPS provides a more stable and centralized tenting effect, which supports both the soft tissue and periosteum during the healing phase. Simultaneous implant placement served a dual role—not only for future prosthetic support but also as a tent-pole structure that enabled vertical grafting without the need for multiple surgeries. Over-grafting above the implant platform was critical for compensating bone remodeling and ensuring long-term graft volume stability and shortened the edentulous healing period [34,35,36]. Because dimensional changes are inevitable during bone remodeling, over-grafting above the implant platform is critical to preserve bone volume. The use of a 2 mm-high tenting pole abutment (SANTA^®^, Biotem) in conjunction with GBR helped maintain vertical space and prevent graft collapse due to soft tissue pressure. Overall, this technique proved to be less complex and resulted in fewer complications than more invasive procedures such as block grafting or titanium mesh. Only one case of partial wound exposure was observed during the follow-up period. This limited complication rate may be attributed to the relatively short observation time and the patients’ adherence to postoperative instructions, particularly careful protection of the surgical site. Moreover, it significantly reduced total treatment duration and the number of surgical interventions, offering a patient-centered, efficient alternative for vertical ridge augmentation [37,38]. Although the present study showed favorable results with minimal complication, it is essential to interpret the findings within the context of its limitations. Similar techniques, such as titanium mesh or mini tenting screws, have reported long-term complications including membrane exposure, screw loosening, graft collapse, and soft tissue dehiscence in follow-up periods extending beyond one year [18,22,23,24,30]. These complications, while infrequent, underscore the need for long-term follow-up to assess the biological stability and prosthetic success of the WHTPS technique over time. Furthermore, several limitations must be acknowledged. First, the small sample size (*n* = 13) reduces the statistical power of this retrospective study and prevents robust subgroup analyses. Although non-parametric tests were employed, the findings should be interpreted cautiously. Second, while smokers were not excluded, no stratified analysis was performed to evaluate the potential impact of smoking on healing outcomes, which may introduce bias or variability in the results. Third, this study lacks a control group for direct comparison with other established vertical augmentation techniques such as narrow-head tenting screws, titanium mesh, and staged GBR. While the preliminary outcomes are promising, comparative data are necessary to assess the true clinical value of WHTPS. To address these issues, a multicenter, randomized controlled trial is currently in development. This future study will include predefined control groups, stratified subpopulations, and standardized outcome metrics such as vertical bone gain, complication rates, and prosthetic success over 6, 12, and 24 months of follow-up. Power analysis will be conducted a priori to ensure sufficient sample size and statistical rigor.

## 5. Conclusions

The WHTPS technique offers a simplified and clinically effective solution for vertical ridge augmentation, particularly in severely resorbed ridges where simultaneous implant placement is either risky or contraindicated. By stabilizing the graft with a single device and supporting over-grafting above implant platforms, this method reduces surgical complexity and facilitates early prosthetic rehabilitation. While the present study provides promising preliminary data, further controlled clinical trials with larger sample sizes, longer follow-up, and comparative analyses are essential to establish the long-term efficacy and predictability of the WHTPS approach in clinical practice.

## Figures and Tables

**Table 1:** Radiographic assessment of post operative outcomes.

Patient	Post-Op Height (mm)	Prosthetic Loading (mm, Month)	Functional Loading (mm, Month)	Bone Gain (mm)	Bone Resorption (mm)
1	29.57	28.91 (5 m)	27.87 (11 m)	11.95	1.7
2	25.82	25.3 (4 m)	24.1 (8 m)	13.06	1.72
3	32.86	32.63 (3 m)	32.43 (8 m)	13.15	0.43
4	16.52	15.8 (4 m)	15.68 (16 m)	10.10	0.84
5	25.33	24.06 (3 m)	23.68 (14 m)	5.47	1.65
6	23.97	21.56 (6 m 3 w)	21.06 (18 m)	7.8	2.91
7	30.20	29.13 (6 m)	29.1 (10 m)	6.03	1.1
8	22.96	22.12 (6 m)	21.64 (20 m)	6.59	1.32
9	28.99	27.19 (7 m)	26.65 (13 m)	7.67	2.34
10	29.37	28.19 (7 m)	28.13 (20 m)	8.32	1.24
11	24.20	23.7 (5 m)	22.9 (8 m)	9.17	1.3
12	25.68	25.56 (7 m)	25.4 (16 m)	5.3	0.28
13	23.78	21.69 (7 m)	21.19 (19 m)	5.59	2.59

## Data Availability

The raw data supporting the conclusions of this article will be made available by the corresponding author on request.
